# Leadership styles and transformational leadership skills among nurse leaders in Qatar, a cross‐sectional study

**DOI:** 10.1002/nop2.1636

**Published:** 2023-02-09

**Authors:** Amer Al‐Thawabiya, Kalpana Singh, Badriya Abdulla Al‐Lenjawi, Albara Alomari

**Affiliations:** ^1^ Hamamd Medical city Doha Qatar

**Keywords:** health service administration, leadership, nursing

## Abstract

**Aim:**

There is a continuing need to implement strategies that create opportunities to develop leadership in Qatar, and to build institutions that can produce effective health managers and leaders. The scarcity of information and studies relating to leadership in this major healthcare corporation must be addressed. This article aims to explore nursing leadership styles and transformational leadership skills among nursing leaders, in Qatar. The study was conducted from October 2020 to January 2021.

**Design:**

A cross‐sectional Study.

**Methods:**

A validated survey was administered to explore the prevalence of leadership styles and transformational leadership skills. Subsequent statistical data analysis achieved the research objectives. The Multi‐Factor Leadership Questionnaire (Western Journal of Nursing Research, 1996) was used as an online de‐identified validated questionnaire.

**Results:**

Eighty‐nine nurses completed the survey. The nurse leaders in this study exhibited leadership traits or qualities that confirm transformational leadership. Some nurse leaders also exhibited transactional and autocratic leadership styles. Directors of nursing exhibit higher levels of transformational leadership style than head nurses, while the latter is more likely to manifest an autocratic leadership style. This study indicates that a development roadmap is needed to transform more nursing leaders into transformational leaders, particularly head nurses, and to universally improve transformational leadership skills among all nursing staff members.

## 1 INTRODUCTION

Leadership style is the key element in the progress of increasing healthcare organizational productivity. Healthcare organizations require diverse leadership styles to work effectively to improve the healthcare sector, trusting in qualified leaders who can think differently and dynamically. Effective leadership in the healthcare sector is essential to improve and enhance systems' effectiveness (Hargett et al., 2017). Leadership style has a vigorous role in the quality of nursing care in hospitals, patient safety, and cost‐effectiveness, in line with a shared organizational vision, mission, governance, and empowerment (Zaghini et al., 2020).

There are three main leadership paradigms focused on people management that play a significant role in determining desired outcomes (Huber, 2017). In the feature of the healthcare system, Huber (2017) defined the transformational leadership style as empowering followers with a sense of autonomy and responsibility, which can increase commitment and efficiency. Moreover, transformational leader facilitates growth and translate evidence into practice to achieve organizational goals. Transformational leadership is thus very common in nursing, inspiring and motivating for a robust transformation of the culture and structure of organizations (Robbins & Davidhizar, 2020) and it is considered the best‐chosen style for leaders in the healthcare sector, especially for nurse leaders to mobilize nursing staff to provide optimum services with improved morale and conviction.

The second leadership style is the transactional style. This style could be effective when the employees are under stress and basic needs are to be considered (Hamilton, 2020). As leadership essentially aims to mobilize followers to achieve organizational interests, transactional leadership mainly acknowledges the values of organizations and employees, but views these as fundamentally separate, entailing a transactional exchange between the organization and its employees (Purwanto et al., 2020). Transactional leaders motivate followers with rewards and punishments for achieving or failing to achieve organizational demands (respectively). This style is appropriate for task‐based contexts where employee autonomy, creativity, and innovation are not a priority (Richards, 2020). In contrast, transactional leadership has also been tremendously efficacious in reducing the degree of errors in healthcare (Fletcher et al., 2019).

The last leadership style is the autocratic style, in autocratic leadership, all decisions are made by leaders, and input from subordinate teams is not encouraged. This authoritarian style of leadership directs staff towards specific tasks they must obey (Durmuş & Kırca, 2019). It promotes a highly structured work environment. It works well when high‐directive decisions are taken, and staff are not considered competent enough to provide autonomous input (Chukwusa, 2018). Autocratic leadership is a classical style of management that does not consider group input. Followers may feel discouraged and experience low morale, nursing resentments and not committing wholeheartedly to the organizational mission and vision, with low satisfaction and commitment translating into poor quality performance, which entails the reduced quality of care and patient outcomes in healthcare contexts.

Nursing leaders drive the integration of care and strengthen the quality of services (De Brún et al., 2019). Nurse leaders assure the proper allocation of the workforce and the optimal level of work quality. Aside from providing the best quality of care, nursing leaders have a fundamental duty to achieve optimum patient satisfaction (Falana et al., 2016). Nursing leaders must motivate nursing staff and cope with emergency and adverse events (Gorman, 2019). Nursing leaders are responsible for patient‐oriented outcomes with the best utilization of their skills, and they need to have emotional stability as leaders to drive followers towards success (Oldland et al., 2020).

Numerous studies discussed the importance of leadership styles in nursing and healthcare in general. Durmuş and Kırca (2019) stated that the transformational leadership style is considered the dominant and gold standard style of leadership. In addition, the literature emphasizes enhancing leadership by encouraging nursing expertise through improved staff stability, and reduced turnout (Sfantou et al., 2017). Furthermore, Transformational leadership is highly related to the employment of effective management that creates a culture of patient safety (Seljemo et al., 2020), while transactional and autocratic leadership had a feeble relationship (Hidayah & Fadila, 2019).

In Qatar, there is a lack of studies about the type of nursing leadership styles, that are necessary to build institutions capable of producing effective health managers and leaders. To achieve any healthcare strategy, nurse leaders must be able to make positive, tangible changes to the delivery of care, which may require a selective mix of leadership styles in different contexts (Gorman, 2019). This study explores leadership styles in the largest healthcare organization in Qatar and focuses on transformational leaders, to identify the chances of improving transformational skills and supporting transformational nursing leaders.

## 2 RESEARCH AIM AND OBJECTIVES

The study aims to address the following research question: ‘what are the leadership styles and transformational leadership skills among nurse leaders in Qatar?’. To achieve this, it targets two objectives, namely:

- Assess the aspects of transformational, transactional, and autocratic leadership styles among nurse leaders.
- Assess the transformational leadership skills of nurse leaders

## 3 METHODOLOGY

An online, survey‐based cross‐sectional study design was used in this study from October to December 2020. The study was conducted at eight large referral hospitals in Qatar. Participants were eligible for the study if they held the positions of Director of Nursing (DON) or Head Nurse (HN) and had at least 1 year of experience as managers in their current positions. A total of 275 nurse leaders were eligible to participate in this study.

With a total population of approximately 275 nursing leaders (i.e., the study population), the sample size was calculated using power analysis; thus, the estimated sample size was 161 participants (with a 95% confidence level tolerated, and a 5% acceptable margin of error). As a result, the sample size was 161. To avoid any response bias, the study employed the modern non‐probability sampling design method of voluntary sampling. The 275 eligible nurse leaders were invited to participate (via their official contact information in HMC), and they could decide voluntarily whether or not to be included in the study. To ensure the data is correct and usable, data cleaning was performed and the final valid number of responses that were suitable for analysis were 89 responses.

## 4 THE INSTRUMENT

The Multi‐Factor Leadership Questionnaire (MLQ 5X short) (Avolio et al., 1999) was used as an online de‐identified validated questionnaire. MLQ is one of the most common effective questionnaires to examine the various levels of leadership competencies and style: transformational, transactional or laissez‐faire (Mahdi & Faraj, 2022). MLQ has undergone numerous revisions and the latest version is known as the MLQ‐5X Short Rater Form (Avolio et al., 1999). Furthermore, the MLQ‐5X is the utmost validated tool to assess transactional and transformational leadership (Boamah & Tremblay, 2019). Q survey was used to distribute the instrument to the participants.

The survey includes 11 items (Transformational style 5 items, transactional 2 items and autocratic 5 items) that measure and evaluate key leadership styles (transformational, transactional, and autocratic), with each question about a specific style, and eight questions that assess transformational leadership skills. All questions are scored on a five‐point Likert scale (0 = strongly disagree, 1 = disagree, 2 = neutral, 3 = agree, 4 = strongly agree). The scores of each style are added together and the total is divided by the number of items, yielding a mean score that represents the mean percentage of the leadership style.

## 5 DATA COLLECTION

Participants were invited via email advertisement sent by the lead researcher, a senior nurse educator, using the official staff email address. The information sheet explained the nature and scope of the study, and the voluntary nature of participation, including the right to withdraw at any time and the right to anonymity. The link to the survey was provided to the participants. The completion of the questionnaire was considered as consent to participate.

## 6 DATA ANALYSIS

To avoid erroneous data analysis results, data were analysed using SPSS version 21 for Windows, including descriptive and inferential analyses. The Shapiro–Wilk test was used to determine the normality of the data distribution; the results showed that the data were not normally distributed. To characterize the demographic features and the MLQ scale scores, descriptive statistics were used. The proposed inferential analysis used the Mann–Whitney U test to assess leadership styles among Qatar directors and HN, and differences in transformational leadership skills between DON and HN.

## 7 ETHICS

There was no official relationship between the researcher and the participants. The Institutional Review Board granted Research Ethics Committee approval (Approval number MRC‐01‐20‐915) 1 month before the study. All data collected through the survey were anonymous and were kept strictly confidential. Upon completion of the survey, the data was stored on Q survey and then it was downloaded and securely stored in the Personal Vault of the HMC cloud storage service provided through Microsoft OneDrive; this service meets the main elements of data security and confidentiality requirements and has been recently utilized to allow access‐controlled collaboration with partners in and outside HMC. The anonymous data is password‐protected and is only accessible to the researcher. To prevent and protect against loss or corruption, the researcher is accountable for ensuring data are backed up frequently.

## 8 RESULTS

In total, 170 nursing staff completed the survey (response rate 62%). Eighty‐nine responses were excluded because the questionnaire forms were not fully completed. The final valid number of responses suitable for data analysis was 89. The majority of participants are serving as HN (60%). The majority of participants had 6–10 years of Qatar experience (21%). Most participants (69%) had been in their current position for 1–5 years. Table 1 provides the characteristics of the participants.

**TABLE 1:** Demographic characteristics.

	*N*	%
Gender		
Female	37	46%
Male	44	54%
Current position at Qatar		
DON	32	40%
HN	49	60%
Years of experience in Qatar		
1–5	9	11%
6–10	17	21%
11–15	17	21%
16–20	37	46%
Years of experience in current position		
1–5	56	69%
6–10	6	7%
11–15	1	1%
16–20	18	22%

### 8.1 Transformational, transactional, and autocratic leadership styles among nurse leaders

**FIGURE 1:**
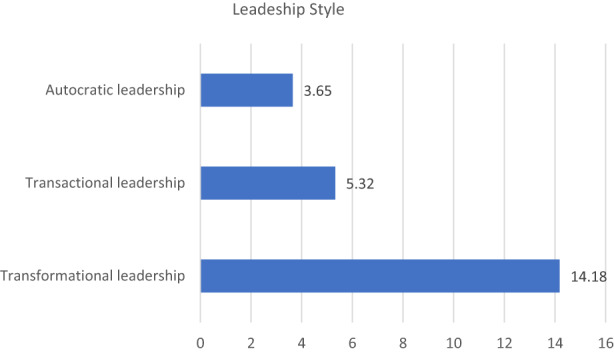
Mean distribution of sample according to their leadership style.

The result shows that the transformational leadership style is the predominantly practised style by nursing leaders (Mean 14.18), although both transactional and autocratic styles are also widely practised (5.32 and 3.65 respectively) as shown in Figure 1.

The result shows that DON's are using more transformational (15.6 ± 4.7), transactional styles (5.7 ± 1.9) and autocratic styles (4.3 ± 3.4) than HN (13.7 ± 6.1; 5.3 ± 2.4 and 3.0 ± 2.1 respectively) which is not statistically significant (Table 2). The autocratic style remains the least used leadership style by both groups.

**TABLE 2:** Results summary.

Leadership style	Current position at Qatar	*N*	Mean ± SD	Median (IQR)	*p* Value
Transformational	DON	32	15.6 ± 4.7	16.5 (14.5, 19.0)	0.16
	HN	49	13.7 ± 6.1	15.0 (10.0, 18.0)	
Transactional	DON	32	5.7 ± 1.9	6.0 (4.0, 7.0)	0.57
	HN	49	5.3 ± 2.4	6.0 (4.0, 7.0)	
Autocratic	DON	32	4.3 ± 3.4	3.0 (2.0, 5.0)	0.15
	HN	49	3.0 ± 2.1	3.0 (2.0, 4.0)	

Abbreviations: DON, Director of Nursing; HN, Head Nurse.

There was no statistically significant difference between gender and years of experience in any of the leadership styles. See Table 3.

**TABLE 3:** Leadership style with years of experience.

	Variables	*N*	Mean ± SD	Median (IQR)	*p* Value
Transformational leadership style					
Years of Experience in HMC	1–5	20	3.92 ± 1.30	15.0 (14.0, 20.0)	0.820
	6–10	35	3.97 ± 0.83	15.0 (14.0, 20.0)	
	11–15	15	3.93 ± 1.01	15.0 (14.0, 20.0)	
	16–20	19	4.19 ± 0.95	15.0 (14.0, 20.0)	
Gender	Male	44	15.0 ± 4.6	16.0 (15.0, 18.0)	0.39
	Female	37	15.4 ± 5.2	17.0 (14.0, 19.0)	
Transactional leadership style					
Years of Experience in HMC	1–5	20	3.91 ± 1.28	7.0 (6.0, 7.0)	0.981
	6–10	35	3.79 ± 0.89	6.0 (4.0, 7.0)	
	11–15	15	3.83 ± 1.03	6.0 (4.0, 7.0)	
	16–20	19	3.84 ± 1.03	6.0 (4.0, 7.0)	
Gender	Male	44	5.7 ± 1.6	6.0 (5.0, 7.0)	0.72
	Female	37	5.6 ± 2.2	6.0 (4.0, 7.0)	
Autocratic leadership style					
Years of Experience in HMC	1–5	20	3.25 ± 1.10	10.0 (6.0, 11.0)	0.784
	6–10	35	3.14 ± 0.81	8.0 (7.0, 10.0)	
	11–15	15	3.30 ± 1.08	9.0 (7.0, 12.0)	
	16–20	19	3.42 ± 0.99	8.0 (7.0, 10.0)	
Gender	Male	44	8.9 ± 3.0	9.0 (7.0, 10.0)	0.53
	Female	37	8.5 ± 2.9	8.0 (7.0, 10.5)	

### 8.2 Transformational leadership skills of nurse leaders

**FIGURE 2:**
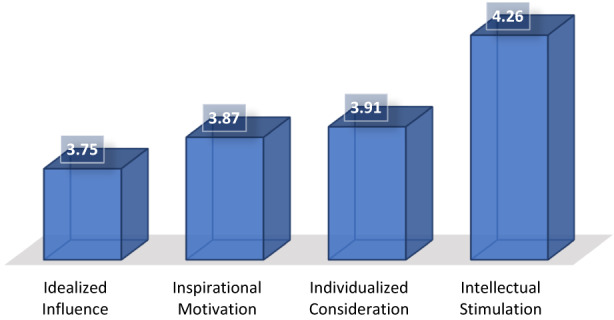
Mean distribution of transformational leadership skills.

Intellectual stimulation is the most prevalent of transformational leadership style skills (Mean of 4.26) among nurse leaders, whereas idealized influence is the least prevalent skill in the group (Figure 2). Individual consideration with a mean of 3.91, followed by 3.87 for inspirational motivation, and idealized influence with a mean of 3.75 is the least prevalent of the four leadership style skills among the population of the study, See Figure 2.

## 9 DISCUSSION

There has been a lack of studies exploring the leadership styles and skills among nurses in Qatar. This study aims to identify the levels of three main leadership styles among nursing leaders in Qatar. Transformational leadership was found to be the most common style among nursing leaders in Qatar. The same results were reported by another recent study which revealed that the leaders rated themselves as transformational more than transactional (Alfadhalah & Elamir, 2019).

The leaders in the current study had higher transformational leadership scores than did leaders in a study conducted in Kuwait (mean 3.3) (Alfadhalah & Elamir, 2019) and higher than did leaders in Turkey (mean 3.90) (Gumusluoglu & Ilsev, 2009). Leaders in the current study tend to use a transformational style more often than transactional or autocratic styles. A recent comprehensive review showed that transformational leaders, understand their followers' needs, build a supportive environment and then engage the followers in practices that instill confidence in them (Hughes et al., 2018).

The study shows that directors of nursing are using a more transformational style than HN but not statistically significant. The result of this study is consistent with a previous cross‐sectional study that showed that HN scored significantly lower (*p* < 0.001) on transformational and transactional leadership styles and significantly higher (*p* < 0.001) on passive‐avoidant leadership styles (Poels et al., 2020). However, the Poels et al. (2020) showed consistent results with the current study that HN's are using an autocratic leadership style than a transformational style. The result of this study is alarming as Collins et al. (2019) stated that, health outcomes are absolute with the transformation style of leadership (Collins et al., 2019). It is considered the best‐chosen style for leaders in the healthcare sector, especially for nurse leaders to mobilize nursing staff to provide optimum services with improved morale and conviction (Morris, 2020). Such leaders are inspired to serve the community in times of distress (Giddens, 2018). Enwereuzer and colleagues reported a positive relationship between transformational style and the engagement of nurses more actively in decision‐making (Enwereuzor et al., 2018). Likewise, Deschamps in his study reported that staff motivation is influenced by transformational style (Deschamps et al., 2016).

The autocratic leadership style was more common among HN could be due to the limitation of the job description, limited delegation and lack of training (Abdul‐Aziz et al., 2020). Another study supports this, stating that the most common reason HN has demonstrated an autocratic style is that it often seems to be confused with administrative positions (Zaccaro et al., 2018). Therefore, HN may not be able to lead staff if their available time is filled with administrative tasks that hinder a visible presence in their wards (Poels et al., 2020). Consequently, the low scores on leadership outcomes may reflect some dissatisfaction of staff with the current ‘absence’ and ‘avoidance’ of leadership. On the same line HN likes to keep possession of the authority of decision‐making in their department (Henen et al., 2018) because they believe that if they do not do so, they would lose control of staff nurses.

About transformational leadership skills, the study illustrated that all skills were present among DON's. However, intellectual stimulation is the most common transformational leadership skill exhibited in nursing leadership. This is supported by Kim and Kim (2017) in their study, 69 senior managers reported that transformational leadership is positively correlated with emotional intelligence (*p* < 0.01) with intellectual stimulation having the strongest correlation with emotional intelligence total scores (*p* < 0.01) (Kim & Kim, 2017). Another study conducted by Kim and Jeong (2020), among 177 managers sought to explore the perception of nurse managers on the application of intellectual stimulation (Kim & Jeong, 2020). The managers rated themselves higher than staff nurses about their application of intellectual stimulation and identified the positive outcome from this transformational leadership factor. It is evident that transformational nurse leaders are effective in terms of stimulating cognitive analysis of problems among their followers and motivating them towards resolving problems more creatively (Al‐Husseini et al., 2021).

Nurse leaders exhibited idealized influence, but at a lower level than intellectual stimulation. This result may require the most attention with respect to creating more transformational leaders. Idealized influence is a skill of transformational leadership whereby leaders share and communicate the organizational vision with followers and actively solicit their feedback to develop more comprehensive and holistic solutions. As Mokhber et al. (2015) stated, this skill enables the leaders to influence and motivate team members because it shows that the leader values team members are enablers of the vision, and responsible for making success possible (Mokhber et al., 2015). In a high‐pressure healthcare environment, where high‐quality services are critically important, idealized influence is an important skill because leaders must be able to motivate and inspire employees towards the common goals of delivering high‐quality service and satisfaction to patients.

## 10 LIMITATIONS

This study used a convenience sampling method to recruit participants and is limited in its generalizability Self‐reporting questionnaires may contain social desirability bias, which refers to participants' proclivity to present a more favourable image of themselves (Van de Mortel, 2008). No information about the influences of the ethnicity of the participants or their time in ranks on the leadership style of the participants was explored in this study.

## 11 RECOMMENDATIONS

The present study seeks to provide baseline data for future studies in determining leadership styles, and organizational culture in Qatar. Therefore, providing training regularly for hospital leaders to improve transformational behaviour and empower the concept of a transformational culture is essential. In addition, selecting people for managerial positions in health care must require their satisfactory participation in accredited leadership training programs. Health organizations should encourage leaders to share governance and provide them with the authority needed to support them in practising transformational leadership. Moreover, greater investment in research is needed to understand how to build transformational leaders and how transformational leadership can be more effective on organizational outcomes. Finally, hospitals should conduct workshops and courses by researchers to explore the effects of transformational style on organizational outcomes. Consultation with followers increases the engagement of the latter with the organizational strategy and goals and inspires trust, respect, and motivation in the team members. A recommended mechanism could be regular, semi‐formal team catch‐up sessions, where leaders discuss their visions with employees and openly listen to and incorporate their input, empowering them to be able to contribute towards delivering the vision. For nursing leaders who exhibited a lower level of skills associated with transformational leadership styles, the amount of development needed is much higher to transform them into transformational leaders. To this end, some areas for a learning and development program must be developed to train nursing leaders with transactional or autocratic leadership styles. Leadership styles, and transformational leadership styles, in particular, remain an under‐researched area in Qatar. Further academic research is required to explore the transformational leadership style prevalence and the benefits it could offer throughout an organization. Finally, Qatar employed international nurses with different backgrounds, further research should explore the relationship between leadership styles and different ethnicity.

## 12 CONCLUSION

This research study was conducted to identify the leadership styles and transformational leadership skills among nurse leaders in Qatar. The transformational leadership style was the most commonly exhibited style among the participants, and the results of the analysis clearly showed it was being practised among the nurse leaders. The study identified a need to promote transformational leadership development in Qatar. DON was identified as being more transformational in style than HN. In terms of transformational leadership skills, except for the individualized consideration, all other skills were present at higher levels among directors of nursing. However, the relative levels in the roles were similar, without any statistically significant difference.

## CONFLICT OF INTEREST STATEMENT

The authors declare that they have no competing interest.

## FUNDING INFORMATION

This project did not receive any specific grant from funding agencies in the public, commercial, or not‐for‐profit sectors.

## Data Availability

Data available on request from the authors.
